# Characterizing the Exercise Behaviour, Preferences, Barriers, and Facilitators of Cancer Survivors in a Rural Canadian Community: A Cross-Sectional Survey

**DOI:** 10.3390/curroncol28040276

**Published:** 2021-08-19

**Authors:** Jenna Smith-Turchyn, Lisa Allen, Jennifer Dart, Deanna Lavigne, Simran Rooprai, Helen Dempster, Richard Trenholm, Daniel Santa Mina, Catherine M. Sabiston, Lindsay MacMillan, Scott C. Adams

**Affiliations:** 1 School of Rehabilitation Science, McMaster University, 1400 Main Street West, Hamilton, ON L8S 1C7, Canada; smithjf@mcmaster.ca; 2 Huntsville Physicians, South Muskoka and Parry Sound Local Education Groups, 100 Frank Miller Drive, Huntsville, ON P1H 1H7, Canada; lisa.allen@mahc.ca (L.A.); jennifer.dart@mahc.ca (J.D.); deannablavigne@gmail.com (D.L.); 3 School of Interdisciplinary Science, Faculty of Science, McMaster University, 1280 Main Street West, Hamilton, ON L8S 4L8, Canada; rooprais@mcmaster.ca; 4 Northern Ontario School of Medicine, 935 Ramsey Lake Road, Sudbury, ON P3E 2C6, Canada; hdempster@mac.com (H.D.); rich.trenholm@mahc.ca (R.T.); lmacmillan@mahc.ca (L.M.); 5 Faculty of Kinesiology and Physical Education, University of Toronto, 55 Harbord Street, Toronto, ON M5S 2W6, Canada; daniel.santamina@utoronto.ca (D.S.M.); catherine.sabiston@utoronto.ca (C.M.S.); 6 Department of Cardiology, Toronto General Hospital Research Institute, 200 Elizabeth Street, Toronto, ON M5G 2C4, Canada; 7 Ted Rogers Cardiotoxicity Prevention Program, Peter Munk Cardiac Centre, 661 University Avenue, Toronto, ON M5G 1X8, Canada

**Keywords:** exercise, barriers, motivators, oncology, rural population

## Abstract

Barriers, facilitators, and motivators to exercise for cancer survivors living in urban settings are well described in the literature. However, there is a lack of comparable information for cancer survivors living in rural communities. We describe the exercise behaviours, barriers, facilitators, and motivators to exercise participation of cancer survivors living in a rural Canadian community. Adult cancer survivors with a primary address in a rural region of Ontario, Canada, who had visited a community hospital in the previous five years were mailed a cross-sectional survey assessing current exercise volume (minutes of moderate-to-vigorous aerobic and resistance exercise), as well as exercise preferences, barriers, and facilitators. Seventy-two survivors (mean age 65 years) completed the survey (16% response rate). A majority of respondents were diagnosed with breast cancer (49%) in the last 5 years (61%). Aerobic- and resistance-training guidelines for cancer survivors were met by 38% and 10% of respondents, respectively. Physical side effects were the most common barrier to exercise during treatment (65%) and post-treatment (35%). Being unaware of available exercise programs, time for exercise, distance to exercise services, and cost were commonly reported barriers during and post-treatment (reported by 10–22%). Respondents reported needing information from a qualified exercise professional (46%), access to a gym (33%) and exercise equipment (26%), and social support (25%) to facilitate exercise participation. Consistent with urban-based cancer survivors, most rural survivors surveyed in this study were not meeting the physical-activity guidelines and reported numerous exercise barriers. These findings can serve as a resource for this and similar rural communities when developing community-based exercise-support services for cancer survivors.

## 1. Introduction

Rural populations are defined as areas that have low population density that are dispersed over a large area [1,2]. Individuals living in rural and remote communities are less active than their urban counterparts [3,4]. Survivors of cancer living in rural and remote communities are considered ‘hard-to-reach’ due to their geographical location; they engage less in cancer survivorship programming; and they have low overall health status, well-being, and quality of life [5].

Cancer survivors are at an increased risk of secondary comorbidities due to direct (e.g., treatment toxicities) and indirect (e.g., secondary deconditioning) cancer treatment-related factors [6]. Notably, acute-treatment-related physiological (e.g., cardiovascular disease and musculoskeletal impairment), psychological (e.g., depression and anxiety), and multi-factorial (e.g., cancer-related fatigue) sequelae can persist for decades after treatment [7,8,9]. If unaddressed, these complications can result in chronic functional impairments, poor health-related quality of life (HRQOL), [10] and, ultimately, premature mortality [11].

In non-cancer settings, exercise is a cornerstone of preventing and treating chronic conditions, as it augments the structure, function of, and coordination between multiple body organs and systems [12,13]. In oncology, findings from systematic reviews and meta-analyses of randomized controlled trials (RCTs) provide strong evidence that exercise training improves a range of physiological (e.g., muscle strength [14] and cardiorespiratory fitness [15]) and patient-reported (e.g., HRQoL [16] and fatigue [17]) outcomes during and following treatment. Moreover, emerging observational evidence suggests that greater levels of engagement in exercise following a cancer diagnosis are associated with significantly reduced cancer-specific and all-cause mortality [18,19,20]. These data provide compelling evidence that exercise may be an effective non-pharmaceutical therapeutic option to prevent and reverse cancer-treatment-related sequalae and prolong life in cancer survivors. Based on this and the related data, exercise is endorsed by leading regional (e.g., Cancer Care Ontario) [21] and national (e.g., American College of Sports Medicine (ACSM)) [22] organizations for cancer survivors during and following treatment.

Unfortunately, only a small portion of cancer survivors living in rural and remote communities take part in regular exercise [3,4]. One factor limiting exercise participation within general populations living in rural communities is that there is greater distance between where people live and where they might exercise [23]. Rural communities may also lack the specialized professionals and dedicated infrastructure needed to deliver evidence-based exercise-support services for cancer survivors [24]. Relatedly, survivors’ medical, physical, and psychological characteristics are highly heterogeneous, which makes it challenging to assess and manage exercise-related contraindications [24,25,26].

Survey-based needs assessments can be used to help identify key support needs, barriers, and service gaps for survivors that inform policy updates, support system changes, and environmental modifications [27]. Previous needs assessments that have assessed the exercise support preferences, facilitators, and barriers for cancer survivors have largely comprised samples derived from urban settings which may not reflect the experiences of those living in remote and rural communities [28,29,30]. To our knowledge, only two exercise oncology needs assessments specifically sampled individuals living in rural settings, and both were restricted to women with breast cancer [31,32]. Moreover, these needs assessments primarily focused on survivors’ exercise preferences and did not explore barriers and facilitators to exercise participation. Understanding how cancer-related factors and exercise preferences, facilitators and barriers influence exercise engagement in rural-dwelling cancer survivors would help inform interventions targeting this at-risk subgroup. As such, a needs-assessment survey is a valuable first step in developing and implementing strategies to improve exercise behaviour. Therefore, the purpose of this study was to characterize the exercise behaviour, barriers, facilitators, and motivators to exercise participation, and diverse exercise support needs of cancer survivors living in a rural Canadian community.

## 2. Materials and Methods

### 2.1. Study Design

This was a cross-sectional survey study. The study was approved by the Laurentian University Research Ethics Board (ID: 6013841). The current report is compliant with the Strengthening the Reporting of Observational Studies in Epidemiology (STROBE) guidelines [33].

### 2.2. Participants and Recruitment

Potential participants were recruited from the North Simcoe Muskoka (Muskoka) region of Ontario, Canada. This region includes residents of Huntsville and Bracebridge and is approximately two hours north of Toronto. Muskoka has a stable regional population of approximately 60,000. With more than 3000 new cancer diagnoses each year, Muskoka residents have a significantly greater age standardized incident rate (SIR; 576 per 100,000) and a similar 5-year survival rate (63.7%) compared to the provincial average (SIR: 532 per 100,000; 63.9% 5-year survival, respectively) [34,35]. Potential participants were identified via patient-registry at the Huntsville District Memorial Hospital (HDMH). The HDMH provides inpatient and outpatient services to a population of just under 20,000 people in North Muskoka. The catchment for HDMH includes a geographical area west towards Parry Sound (not including Parry Sound), east to Algonquin Park, south to Baysville, and north to South River, Ontario.

All individuals who were over the age of 18, fluent in English, had a pathologically or radiologically confirmed diagnosis of cancer (any type and stage), had a primary address in the Muskoka region, and were seen or treated at HDMH for cancer in the previous five years were invited to take part in this study and were mailed a needs-assessment survey package. To protect patients’ privacy, mailout packages were printed and collated by study staff (DL) prior to being sent to the hospital, where they were addressed, stamped, and mailed by a hospital research administrator. The surveys were collected between May and October 2019—approximately 6 months before the declaration of the Covid-19 pandemic. We used an implied consent process for the survey study wherein participants were informed (via mailout information sheet) that the act of returning the completed questionnaire was interpreted as them having provided consent (Supplementary Questionnaire S1).

### 2.3. Research Instrument

The Needs Assessment Survey included four parts (Part 1, Demographic Information; Part 2, Cancer History; Part 3, Exercise Needs Assessment (including exercise volume (how often and for how long do you take part in (1) strenuous, (2) moderate, and (3) mild exercise?), barriers to exercise during and after treatment, exercise motivators, and needed facilitators); and Part 4, Interest in Community Exercise Study). See Supplementary Questionnaire S1 for a copy of the Needs Assessment Survey used. This 51-question survey was administered in English and took approximately 30 min to complete. Questions included multiple choice, multi-select (where respondents select all that apply), rating, and open-ended questions. No validated exercise oncology needs-assessment survey tools have been developed to date. Thus, the survey used was created by three members of the study team (S.A., J.S.T., and L.A.) and was informed by a previous needs assessment [36], a validated outcome measure assessing exercise volume [37], and research on exercise-related barriers, facilitators, and needs for cancer survivors [25].

### 2.4. Data Collection and Analysis

Surveys were returned via a pre-paid envelope and stored in a secure filing cabinet at HDMH (Huntsville, Ontario). Data from the paper copies of the surveys were manually entered into an Excel spreadsheet (Microsoft^®^ Excel for Mac, Version 16.43 (20110804)). Survey questions were analyzed by using descriptive statistics by reporting frequencies (percentages) and means ± standard deviations, as appropriate. Exploratory analyses were performed to identify demographic, medical, and participant-level factors often corresponding to greater patient support needs and vulnerability (e.g., socioeconomic status, comorbidities, cancer stage, and treatment status) that were associated with differences in the assessed outcomes. Student’s *t*-test, Chi-squared, or Fischer’s Exact analyses were used where appropriate to explore associations between exercise behaviour, barriers, facilitators, and motivators, as well as exploratory demographic, medical, and participant-level factors. STATAICv15 was used to conduct all analyses, with the significance set at *p* < 0.05.

## 3. Results

A total of 72 community members responded to the survey mailouts (of 449 mailouts sent; response rate, 16%). Information regarding respondents’ demographic characteristics and medical history is summarized in Table 1 and Table 2, respectively. Briefly, the majority of respondents (average age 65 ± 10 years) were Caucasian (96%), in relationships (74%), had household incomes of <$60,000/year (54%), were retired (67%), had never smoked (53%), had a diagnosis of breast cancer (49%), were diagnosed less than 5 years ago (61%), had localized disease (63%), had received cancer surgery (88%), chemotherapy (89%) and/or radiotherapy (60%), and were currently post-treatment (68%).

### 3.1. Comorbidities, Symptoms, and Limitations

All but nine respondents (87%) reported at least one other comorbid chronic condition, with the most reported comorbidities being high blood pressure, arthritis, a second cancer diagnosis (each reported in 38% of respondents), and high cholesterol (24%) (Table 1). Fifty-eight percent of respondents reported currently experiencing side effects of cancer treatments, including fatigue (49%), decreased strength (38%), pain (32%), and decreased range of motion (26%; Table 2). The median daily fatigue rating of respondents was 4.5 (moderate fatigue) on a scale of 0 (no fatigue) to 10 (extreme fatigue), and 58% reported a recent change in weight (25% weight loss and 33% weight gain). The majority (63%) of participants reported that their ability to exercise was limited by a health condition, injury, or disability. When asked if their current cancer-related side effects affected their function, 39% reported yes, with 18% saying they were unable to return to important activities at home, 15% saying they were unable to return to important activities in the community, and 6% saying they were unable to return to work.

### 3.2. Exercise Behaviour and Preferences

Only 38% of respondents reported currently meeting the guidelines for aerobic exercise per week (90–150 min of moderate to vigorous exercise per week [21,22]), and 10% of respondents reported meeting resistance training guidelines (two sessions per week [21,22]). The percentage of respondents reporting being somewhat-to-very active varied by treatment stage. Specifically, moderate-to-high levels of activity were reported by 89% of respondents prior to treatment, 40% during treatment, and 63% following treatment. There was a significant difference in the percentage of respondents reporting being very active across treatment stages (pre-, during, and post-treatment; *p* < 0.001). When exploring exercise preferences, 56% of respondents preferred to exercise alone, either at home (44%) or outside (42%). Further analysis found no significant difference in total minutes of weekly moderate-to-vigorous intensity aerobic exercise for any explored variable; however, those reporting fewer exercise limitations reported meeting aerobic exercise guidelines in greater proportion than those reporting more limitations (*p* = 0.04), as did those taking fewer medications (*p* = 0.01), those with localized disease (*p* = 0.02), and those with lower levels of fatigue (*p* = 0.02). Refer to Table 3 for further detail on exercise level of respondents overall and by participant characteristic.

### 3.3. Exercise Interests, Beliefs, and Goals

The majority of respondents (54%) felt it would be beneficial to exercise in general; and 50% of respondents reported they believed that participation in an exercise program could help them with their cancer-related concerns. Most participants (58%) indicated having a goal of increasing their exercise levels. However, respondents reported a mean confidence of 6/10 in their ability to exercise 3–5 times per week for 30–60 min per session. Finally, over half of respondents (53%) said they were currently interested in joining a community exercise program for cancer survivors. Those who believed exercise was beneficial were significantly more likely to express interest in joining a cancer-specific community-based exercise program (*p* < 0.001).

### 3.4. Exercise Barriers, Facilitators, and Motivators

Table 4 and Table 5 present data on exercise barriers, facilitators, and motivators. Briefly, physical side effects were the most commonly reported barrier to exercise during (65%) and following (35%) treatment. During treatment, 10–13% of respondents reported unawareness of an exercise program, time, exercise-program distance, and cost as barriers. Following treatment, unawareness of an exercise program, time, and exercise-program distance remained the next most common barriers, as reported by 13–22% of respondents. In subgroup analyses, a greater proportion of breast cancer survivors reported cost as a barrier to exercise during treatment (*p* = 0.005) compared to survivors of other cancer types, and a greater proportion of respondents with metastatic disease reported physical side effects as a barrier during treatment compared to those with localized disease (*p* = 0.001). Additionally, more respondents with an annual household income ≤ $40,000 reported cost as a barrier to exercise both during treatment (*p* = 0.04) and post-treatment (*p* = 0.04) compared to those with an annual household income > $40,000. Finally, those reporting greater than two side effects reported cost and side effects as barriers to exercise in greater proportions both during treatment (*p* = 0.04 and *p* = 0.009, respectively) and post-treatment (*p* = 0.05 and *p* = 0.01). Refer to Table 4 for more information on barriers to exercise by participant characteristic.

Respondents reported needing information from a qualified exercise professional (QEP) (46%), access to a gym (33%), access to exercise equipment (26%), and social support (25%) to facilitate regular exercise participation. Those with higher levels of fatigue and reporting a greater number of side effects perceived that support from a QEP would facilitate exercise in a greater proportion than those with lower levels of fatigue and fewer side effects (*p* = 0.003 and *p* = 0.054, respectively). Moreover, respondents who perceived exercise to be beneficial for their cancer-related concerns reported access to a gym (*p* = 0.011), access to exercise equipment (*p* = 0.003), social support (*p* = 0.001), and information from a QEP (*p* = 0.004) as facilitators that would support their exercise participation more than those who did not perceive exercise as being beneficial. Finally, a greater proportion of those interested in a community-based exercise program reported access to a gym (*p* = 0.013) and information from a QEP (*p <* 0.001) as things that would facilitate exercise more than those who were not interested a program. Refer to Table 5 for more information on the facilitators to exercise for this population.

When asked about their current motivation to exercise, more than half of participants reported increasing strength (57%), increasing their overall fitness level (53%), and preventing recurrence (51%) as primary motivators to exercise. A greater proportion of those who perceived exercise as beneficial to their current health were more likely to report all tested variables as sources of exercise motivation (*p* < 0.05), as were those who thought exercise was beneficial for their cancer-related concerns. See Table 5 for further details on sources of exercise motivation.

## 4. Discussion

The purpose of this study was to characterize exercise behaviour and determinants within a diverse sample of cancer survivors living in a single rural community in Canada to inform the development of local exercise-support services. Overall, when looking at the findings of this study compared to needs assessments of urban-based cancer survivors, many common barriers (physical side effects, cost of programs, lack of awareness of programs, and time to exercise) and facilitators (access to exercise programs/equipment and QEP support) were reported. Rural cancer survivors surveyed in this study were inactive and, similar to their urban-based counterparts, reported that cancer-related side effects were the most common exercise limitation during and following treatment. Compared to urban-based exercise oncology needs assessments, distance to exercise programming was a unique barrier reported by survey respondents in this study; however, this finding is consistent with reported exercise barriers for the general population living in rural communities [23,24]. Our findings highlight important subgroup-specific differences in exercise barriers, facilitators, and motivators, which can be used to adapt intervention delivery approaches for cancer survivors. Notably and consistent with previous research [38,39,40], positive survivor beliefs regarding the general health and cancer-specific benefits of exercise consistently associated with more sources of exercise motivation, more reported ways to facilitate exercise, and higher interest in participating in local exercise support services. Together, these findings suggest that support interventions designed to improve survivors’ perceptions towards the benefits of exercise may play an important role in improving exercise engagement in cancer survivors.

To our knowledge, this was the first exercise-needs assessment of individuals diagnosed with multiple cancer types (i.e., other than breast cancer) living in a rural community. At 20 to 40% of the current sample, the percentage of survey respondents reporting meeting current physical-activity guidelines for aerobic exercise (90–150 min of moderate-intensity aerobic exercise per week [21,22]) was consistent with previous reported levels of physical activity for cancer survivors in Canada [41]. At 10%, the percentage of respondents reporting meeting current physical-activity guidelines for resistance training (two times per week, at moderate intensity for all major muscle groups [21,22]) was also consistent with urban-dwelling cancer survivors [42]. Our findings indicate that survivors with more severe disease (i.e., those with metastatic disease and those describing higher levels of exercise limitations, side effects, and medication use) were meeting exercise guidelines less frequently. Clearly, additional precautions must be taken when approaching exercise for survivors living with metastatic disease [21] and with multiple comorbidities [26]. However, these survivors arguably stand to benefit most from the protective and restorative effects of exercise. For example, exercise has been shown to improve physical function and HRQOL in survivors living with advanced disease [43] and may even improve the stability of metastatic lesions and normalize the microenvironment [44]. Consistent with previous research [10,11], approximately two-thirds of respondents reported currently living with lingering side effects of treatment, with a third saying that this significantly affected their overall functional level. Respondents who reported a higher number of current side effects (>2) were more likely to report cost and physical side effects as barriers to exercise both during treatment and post-treatment. Notably, persistent functional limitations caused by treatment-related side effects, such as fatigue, upper extremity morbidity, cognitive issues, and depression, are associated with impaired return to work post-treatment [45]. The cessation of work is associated with poor HRQOL [45] and financial strain within survivors [46], and it may further limit their ability to take part in supportive care services, such as exercise programming. Therefore, future research is needed to examine the most effective ways to provide safe and accessible exercise interventions for those living with advanced disease and persistent side effects.

### 4.1. Implications for Practice: What Is Needed?

Similar to previous studies in urban communities, the results of this study demonstrate that cancer survivors living in this rural community need access to a gym, exercise equipment, and information from a QEP to facilitate their exercise participation. Our findings demonstrate a need to provide additional support for individuals who are living with health impairments (e.g., side effects), those who have a more severe disease (e.g., metastatic disease), and those with other chronic health conditions (e.g., multiple comorbidities) to overcome barriers to exercise. Such considerations are vital, as one in four Canadian adults live with two or more chronic conditions, and half of older adults in Canada are living with three or more chronic conditions [47,48]. While it was encouraging that half of respondents were interested in a community-based exercise program for cancer survivors, it must be acknowledged that half were not. From a public-health perspective, these findings highlight a need to devise interventions to improve the attitudes, beliefs, and motivations to exercise in those who are not ready to change their behaviour. Thus, future work is needed to create and disseminate educational materials for cancer survivors in this region who report not currently being interested in exercise programs. Ultimately, providing appropriate and accessible exercise services in this community will help to facilitate sustained exercise behaviour change and the physiological, psychological, and social benefits that go along with that change.

### 4.2. Limitations

The findings of this project should be reviewed with an understanding of its limitations. Firstly, only a small portion of individuals completed and returned the survey (16% response rate). While this response rate is consistent with similar mail-based surveys [49], the limited response rate may have led to a response bias, as only individuals with an interest in this topic may have responded to this survey. Future research should expand recruitment duration and methods to enhance participation. Recruitment methods could include using web-based applications and social-media platforms, as well as recruitment at physician offices and other community-health and cancer-support service locations. Additionally, results of this study are specific to individuals living within a specific rural region of Ontario, Canada. While some rural communities may have similar characteristics, there may also be important differences in the resources (natural, physical, and professional) and support services across regions. Other limiting factors include the majority of respondents being Caucasian and having been diagnosed with breast cancer. Collectively, these factors may limit the generalizability of these results to other ethnicities, cancer types, and rural communities. Finally, while we assessed physical-activity behaviour using a validated questionnaire, the remaining components of the needs assessment have not previously been validated. To our knowledge, no exercise-based needs assessment has been validated in cancer survivors.

## 5. Conclusions

This study provides important insights into exercise behaviour, barriers, facilitators, and motivators of cancer survivors living in a specific rural Canadian region. Findings are consistent with other exercise-needs assessments performed in urban and rural communities. Most survivors were not meeting the exercise guidelines and reported cost-, time-, and distance/transportation-related barriers to exercise. The reported barriers most often differed depending on the number of exercise-facilitating and -motivating factors. Described facilitators to exercise and needs of respondents included access to a QEP, fitness equipment, and fitness centres. Survivors who perceived exercise to be beneficial consistently reported more exercise-facilitating and -motivating factors. Future research is required to expand upon our work in other rural communities and in non-breast cancer rural survivors. However, these findings will serve as a valuable resource for this rural area when developing community-based exercise-support services for cancer survivors.

## Figures and Tables

**Table 1:** Participant demographics (total *n* = 72).

Participant Characteristics	No. ^1^	% ^1^
Age (years): mean (SD)	65 (10.3)	
**Age Range**		
40–49 years	6	8
50–59 years	15	21
60–69 years	17	24
70+ years	30	42
**Residence Location ^2^**		
≤10 min	26	36
20–30 min	28	39
≥40 min	18	25
**Marital Status**		
Never married	3	4
Married/Common law	53	74
Separated/Widowed/Divorced	16	22
**Education Level**		
Some or all high school	25	35
Some or all university	36	50
Some or all grad school	11	15
**Annual Household Income**		
<$60,000	39	54
$60,000–$99,999	15	21
≥$100,000	11	15
**Employment Status**		
Disability/sick leave	8	11
Retired	48	67
Part-Time	4	6
Full-Time	12	17
**Ethnicity**		
Caucasian	69	96
Other	2	3
**Smoking Status**		
Never	38	53
Quit	31	43
Current	2	3
**Comorbidities**		
Angina	6	8
Arthritis	27	38
Cancer (second)	27	38
Chronic bronchitis	4	6
Diabetes	10	14
Heart attack	5	7
High blood pressure	27	38
High cholesterol	17	24
Stroke	4	6
Other	15	21
**Medication Use**		
No medications	18	25
1 medication	17	24
2 medications	15	21
3 medications	7	10
4 medications	6	8
≥5 medications	9	13

^1^ Participant totals and percentages may not add up because some respondents did not answer all questions. ^2^ Driving time from downtown.

**Table 2:** Cancer history.

Medical Characteristics	No. ^1^	% ^1^
**Cancer Type**		
Breast	35	49
Colorectal	9	13
Leukemia	2	3
Lung	5	7
Lymphoma	8	11
Ovarian	4	6
Pancreatic	3	4
Prostate	2	3
Other	4	6
**Date of Diagnosis**		
≤2013	6	8
2014–2015	16	22
2016–2017	24	33
2018–2019	20	28
Not reported	6	8
**Lymph Node Involvement**		
Yes	44	61
No	20	28
Unsure	6	8
**Disease Stage**		
Localized	45	63
Metastatic:	24	33
Bone	3	4
Liver	6	8
Lung	4	6
Lymph Node	18	25
**Cancer Recurrence**		
Yes	9	13
No	57	79
Unsure	6	8
**Treatment Exposure**		
Surgery	63	88
Chemotherapy	64	89
Radiotherapy	43	60
Other	17	24
**Treatment Status**		
Current treatment	18	25
Post treatment	49	68
Not reported	5	7
**Treatment Complications**		
Yes	42	58
No	27	38
Not Reported	3	4
**Current Side Effects**		
Anxiety	13	18
Decreased range of motion	19	26
Decreased strength	27	38
Depression	11	15
Fatigue	35	49
Loss of appetite	8	11
Lymphedema	12	17
Nausea/vomiting	7	10
Pain	23	32
Other	13	18

^1^ Participant totals and percentages may not add up because some respondents did not answer all questions.

**Table 3:** Exercise engagement overall and by subgroups of rural cancer survivors.

	n	Moderate-to-Vigorous Intensity PA (Minutes/Week)					Meeting AET Guidelines			Meeting RET Guidelines		
		Mean	SD	Difference	95% CI	*p*	n	%	*p*	n	%	*p*
**Overall**	72	142.4	263.1				27	38		7	10	
**Subgroups’**												
**Age**												
≤66.5 years	34	119.9	168.7	−61.8	−191.9 to 68.3	0.35	14	21	0.08	4	6	1.0
>66.5 years	34	181.6	340.4				13	19		3	4	
**Income**												
<$40,000/year	26	156.0	345.7	1.7	−137.9 to 141.3	0.98	8	12	0.22	3	5	1.0
≥$40,000/year	39	154.2	218.1				18	28		4	6	
**Exercise Limitations**												
≤2 limitations	42	159.3	190.9	40.6	−85.3 to 166.6	0.52	20	28	**0.04 ^1^**	5	7	0.69
>2 limitations	30	118.7	342.0				7	10		2	3	
**Medication Use**												
≤2 medications	50	162.4	209.1	65.6	−68.7 to 199.9	0.33	24	33	**0.008**	6	8	0.63
>2 medications	22	96.8	359.0				3	4		1	1	
**Fatigue Score (/10)**												
0–2	20	190.3	189.5	26.8	−142.0 to 195.6	0.23 ^2^	13	18	**0.015**	3	4	0.30
3–5	33	163.5	344.9	108.2	−56.0 to 272.4		9	13		4	6	
6–10	19	55.3	110.6				5	7		0	0	
**Cancer Diagnosis**												
Breast cancer	35	134.3	216.8	−15.7	−108.8 to 140.3	0.80	13	18	0.95	3	4	1.0
Other cancers	37	150.0	303.3				14	19		4	6	
**Cancer Stage**												
Local (Stages I–III)	45	180.7	303.7	94.8	−39.1 to 228.8	0.16	22	32	**0.02**	6	9	0.41
Metastatic (Stage IV)	24	85.8	170.0				5	7		1	1	
**Cancer Side Effects**												
≤2 side effects	42	135.7	204.5	−16.0	−142.2 to 110.3	0.80	17	24	0.54	7	10	**0.04**
>2 side effects	30	151.7	332.0				10	14		0		
**Perceived Exercise Benefit:** **General Health**												
No/Unsure	30	148.3	335.6	−0.4	−130.8 to 130.0	1.0	9	13	0.17	3	4	1.0
Yes	39	148.7	204.2				18	26		4	6	
**Perceived Exercise Benefit:** **Cancer-Related**												
No	20	194.0	396.4	75.9	−85.3 to 237.2	0.35	7	13	0.90	2	4	1.0
Yes	36	118.1	207.5				12	21		4	7	
**Current PA Goals**												
No Increase in PA	25	204.8	354.7	86.9	−48.9 to 222.7	0.21	12	18	0.23	4	6	0.41
Increase in PA	42	117.9	203.0				14	21		3	4	
**Current Exercise Motivators**												
≤3 motivators	39	118.9	291.4	−51.3	−175.7 to 73.0	0.41	11	15	0.08	2	3	0.24
>3 motivators	33	170.2	226.4				16	22		5	7	
**Exercise Facilitating Factors**												
≤1 facilitator required	43	141.6	292.6	−1.8	−128.8 to 125.2	0.98	15	21	0.58	3	4	0.43
>1 facilitator required	29	143.5	217.0				12	17		4	6	
**Interest in Community Exercise Program**												
No/Unsure	31	150.2	321.7	2.9	−127.1 to 132.9	0.96	10	14	0.29	4	6	0.69
Yes	38	147.2	217.3				17	25		3	4	

^1^ Bolded values represent significant differences between subgroups; ^2^ Comparison between all three groups using one-way ANOVA.

**Table 4:** Reported barriers to exercise during and following treatment for rural cancer survivors.

	Barriers to Exercise During Treatment														Barriers to Exercise Following Treatment													
	Cost		Program Awareness		Time		Program Distance		Child Care		Transport		Side Effects		Cost		Program Awareness		Time		Program Distance		Child Care		Transport		Side Effects	
	n	(%)	n	(%)	n	(%)	n	(%)	n	(%)	n	(%)	n	(%)	n	(%)	n	(%)	n	(%)	n	(%)	n	(%)	n	(%)	n	(%)
**Overall**	7	(10)	9	(13)	9	(13)	9	(13)	3	(4)	4	(6)	47	(65)	11	(6)	16	(22)	9	(13)	9	(13)	3	(4)	4	(6)	25	(35)
**Subgroups’**																												
**Age**																												
≤66.5 years (n = 33)	4	(12)	4	(12)	2	(6)	3	(9)	3	(9)	3	(9)	25	(76)	6	(19)	9	(28)	5	(16)	4	(13)	3	(9)	**4 ^1^**	**(13)**	14	(44)
>66.5 years (n = 34)	2	(6)	4	(12)	6	(18)	5	(15)	0	(0)	0	(0)	20	(59)	4	(12)	6	(18)	4	(6)	5	(15)	0	(0)	**0**	**(0)**	10	(29)
**Income**																												
<$40,000/year (n = 26)	**5**	**(19)**	3	(12)	6	(23)	6	(23)	2	(8)	3	(12)	16	(62)	**7**	**(29)**	5	(21)	6	(25)	6	(25)	2	(8)	3	(13)	10	(42)
≥$40,000/year (n = 38)	**1**	**(3)**	5	(13)	3	(8)	3	(8)	1	(3)	1	(3)	28	(74)	**3**	**(8)**	10	(26)	3	(8)	3	(8)	1	(3)	1	(3)	14	(37)
**Exercise Limitations**																												
≤2 limitations (n = 41)	4	(10)	**2**	**(5)**	**2**	**(5)**	6	(15)	0	(0)	1	(2)	24	(59)	6	(15)	6	(15)	5	(13)	6	(15)	0	(0)	1	(3)	**10**	**(25)**
>2 limitations (n = 30)	3	(10)	**7**	**(23)**	**7**	**(23)**	3	(10)	3	(10)	3	(10)	23	(77)	5	(17)	10	(35)	4	(14)	3	(10)	3	(10)	3	(10)	**15**	**(52)**
**Medication Use**																												
≤2 medications (n = 49)	6	(12)	5	(10)	**3**	**(6)**	5	(10)	**0**	**(0)**	1	(2)	32	(65)	7	(15)	10	(63)	5	(10)	6	(13)	**0**	**(0)**	2	(4)	16	(33)
>2 medications (n = 22)	1	(5)	4	(18)	**6**	**(27)**	4	(18)	**3**	**(14)**	3	(14)	15	(68)	4	(19)	6	(38)	4	(19)	3	(14)	**3**	**(14)**	2	(10)	9	(43)
**Fatigue Score/10**																												
0–2 (n = 19)	1	(5)	1	(5)	2	(11)	4	(21)	0	(0)	1	(5)	9	(47)	4	(22)	3	(17)	2	(11)	3	(17)	0	(0)	0	(0)	**1**	**(6)**
3–5 (n = 33)	3	(9)	3	(9)	3	(9)	3	(9)	1	(3)	1	(3)	22	(31)	3	(9)	6	(19)	3	(9)	3	(9)	1	(3)	2	(6)	**12**	**(38)**
6–10 (n = 19)	3	(16)	5	(26)	4	(21)	2	(11)	2	(11)	2	(11)	16	(84)	4	(21)	7	(37)	4	(21)	3	(16)	2	(11)	2	(11)	**12**	**(63)**
**Cancer Diagnosis**																												
Breast cancer (n = 35)	**7**	**(20)**	4	(11)	4	(11)	5	(14)	2	(6)	3	(9)	21	(60)	9	(26)	5	(14)	4	(11)	6	(17)	2	(6)	3	(9)	12	(34)
Other cancers (n = 36)	**0**	**(0)**	5	(14)	5	(14)	4	(11)	1	(3)	1	(3)	26	(72)	2	(6)	11	(32)	5	(15)	3	(9)	1	(3)	1	(3)	13	(38)
**Cancer Stage**																												
Local (Stages I–III) (n = 44)	5	(11)	5	(11)	6	(14)	5	(11)	2	(5)	2	(5)	**25**	**(56)**	9	(21)	10	(24)	6	(14)	3	(7)	2	(5)	2	(5)	14	(33)
Metastatic (Stage IV) (n = 24)	2	(8)	4	(17)	3	(13)	4	(17)	1	(4)	2	(8)	**21**	**(88)**	2	(8)	6	(25)	3	(13)	5	(21)	1	(4)	2	(8)	11	(46)
**Cancer Side Effects**																												
≤2 side effects (n = 41)	**1**	**(2)**	4	(10)	6	(15)	6	(15)	1	(2)	1	(2)	**22**	**(54)**	**3**	**(8)**	6	(15)	5	(13)	6	(15)	1	(3)	**0**	**(0)**	**9**	**(23)**
>2 side effects (n = 30)	**6**	**(20)**	5	(17)	3	(10)		(10)	2	(7)	3	(10)	**25**	**(83)**	**8**	**(27)**	10	(33)	4	(13)	3	(10)	2	(7)	**4**	**(13)**	**16**	**(53)**
**Perceived Exercise Benefit: General Health**																												
No/Unsure (n = 30)	2	(7)	3	(10)	2	(7)	2	(7)	0	(0)	1	(3)	21	(70)	2	(7)	6	(20)	**1**	**(3)**	3	(10)	0	(0)	2	(7)	9	(30)
Yes (n = 38)	5	(13)	6	(16)	6	(16)	6	(16)	3	(8)	2	(5)	26	(68)	9	(24)	10	(27)	**8**	**(22)**	6	(16)	3	(8)	2	(5)	16	(43)
**Perceived Exercise Benefit: Cancer-Related**																												
No (n = 19)	1	(5)	1	(5)	1	(5)	2	(11)	0	(0)	1	(5)	13	(68)	1	(5)	4	(21)	1	(5)	3	(16)	0	(0)	1	(5)	6	(32)
Yes (n = 36)	6	(17)	7	(19)	5	(14)	5	(14)	3	(8)	2	(6)	27	(75)	10	(29)	11	(31)	7	(20)	5	(14)	3	(9)	3	(9)	16	(46)
**Current PA Goals**																												
No increase in PA (n = 24)	**0**	**(0)**	1	(4)	1	(4)	2	(8)	0	(0)	0	(0)	17	(71)	**1**	**(4)**	4	(17)	1	(4)	3	(13)	0	(0)	0	(0)	6	(25)
Increase in PA (n = 42)	**7**	**(17)**	7	(17)	7	(17)	6	(14)	3	(7)	3	(7)	30	(71)	**10**	**(24)**	12	(29)	8	(20)	6	(15)	3	(7)	4	(10)	19	(46)
**Current Exercise Motivators**																												
≤3 motivators (n = 38)	**1**	**(3)**	4	(11)	3	(8)	4	(11)	0	(0)	2	(5)	24	(63)	3	(8)	7	(19)	3	(33)	4	(11)	0	(0)	1	(3)	**9**	**(24)**
>3 motivators (n = 33)	**6**	**(18)**	5	(15)	6	(18)	5	(15)	3	(9)	2	(6)	23	(70)	8	(25)	9	(28)	6	(67)	5	(16)	3	(9)	3	(9)	**16**	**(50)**
**Exercise Facilitating Factors**																												
≤1 facilitator required (n = 41)	3	(7)	**2**	**(5)**	**2**	**(5)**	5	(12)	0	(0)	2	(5)	24	(59)	**2**	**(5)**	**5**	**(13)**	**2**	**(5)**	5	(13)	0	(0)	1	(3)	**9**	**(23)**
>1 facilitator required (n = 30)	4	(13)	**7**	**(23)**	**7**	**(23)**	4	(13)	3	(10)	2	(7)	23	(77)	**9**	**(30)**	**11**	**(37)**	**7**	**(23)**	4	(13)	3	(10)	3	(10)	**16**	**(53)**
**Interest in Community Exercise Program**																												
No/Unsure (n = 31)	1	(3)	1	(3)	1	(3)	1	(45)	0	(0)	2	(7)	17	(55)	2	(7)	4	(13)	1	(3)	5	(16)	0	(0)	2	(7)	7	(23)
Yes (n = 38)	6	(16)	7	(18)	6	(16)	4	(11)	3	(8)	1	(3)	30	(79)	8	(21)	12	(32)	7	(18)	4	(11)	3	(8)	2	(5)	18	(47)

^1^ Bolded values represent significant differences between subgroups.

**Table 5:** Exercise-facilitating and -motivating factors for rural cancer survivors.

	Exercise Facilitating Factors										Exercise Motivations															
	Gym Access		Equipment Access		Social Support		Transport		QEP Information		Increase Fitness		Reduce Stress		Social Interactions		Prevent Disease		Weight Loss		Do Things That Matter		Prevent Recurrence		Increase Strength	
	n	(%)	n	(%)	n	(%)	n	(%)	n	(%)	n	(%)	n	(%)	n	(%)	n	(%)	n	(%)	n	(%)	n	(%)	n	(%)
**Overall**	24	(33)	19	(26)	18	(25)	3	(4)	33	(46)	38	(53)	31	(43)	21	(29)	25	(35)	28	(39)	26	(36)	37	(51)	41	(57)
**Subgroups’**																										
**Age**																										
≤66.5 years (n = 34)	10	(31)	9	(28)	9	(28)	2	(6)	18	(56)	21	(62)	17	(50)	9	(27)	14	(41)	16	(47)	9	(27)	17	(50)	21	(62)
>66.5 years (n = 34)	13	(39)	9	(27)	8	(24)	1	(3)	14	(42)	15	(44)	13	(38)	11	(32)	10	(29)	10	(29)	16	(47)	20	(59)	18	(53)
**Income**																										
<$40,000/year (n = 26)	7	(30)	6	(26)	6	(26)	1	(4)	**7 ^1^**	**(30)**	12	(48)	14	(56)	10	(40)	11	(44)	**6**	**(24)**	9	(36)	15	(60)	15	(60)
≥$40,000/year (n = 38)	16	(42)	11	(29)	11	(29)	2	(5)	**24**	**(63)**	24	(62)	15	(39)	10	(26)	13	(33)	**19**	**(49)**	15	(39)	20	(51)	23	(59)
**Exercise Limitations**																										
≤2 limitations (n = 41)	13	(33)	11	(28)	10	(26)	2	(5)	16	(41)	24	(57)	17	(41)	11	(26)	15	(36)	15	(36)	12	(29)	21	(50)	23	(55)
>2 limitations (n = 30)	11	(38)	8	(28)	8	(28)	1	(3)	17	(59)	14	(48)	14	(48)	10	(35)	10	(35)	13	(45)	14	(48)	16	(55)	18	(62)
**Medication Use**																										
≤2 medications (n = 49)	15	(32)	11	(23)	12	(26)	2	(4)	24	(51)	30	(60)	22	(44)	14	(28)	19	(38)	19	(38)	16	(32)	27	(54)	29	(58)
>2 medications (n = 22)	9	(43)	8	(38)	6	(29)	1	(5)	9	(43)	8	(38)	9	(43)	7	(33)	6	(29)	9	(43)	10	(48)	10	(48)	12	(57)
**Fatigue Score/10**																										
0–2 (n = 19)	6	(33)	4	(22)	2	(11)	1	(6)	**7**	**(39)**	10	(53)	7	(37)	3	(16)	6	(32)	5	(26)	**4**	**(21)**	8	(42)	9	(47)
3–5 (n = 33)	12	(39)	10	(32)	9	(29)	2	(7)	**12**	**(39)**	15	(46)	14	(42)	9	(27)	13	(39)	17	(52)	**11**	**(33)**	18	(55)	17	(52)
6–10 (n = 19)	6	(32)	5	(26)	7	(37)	0	(0)	**14**	**(74)**	13	(68)	10	(53)	9	(47)	6	(32)	6	(32)	**11**	**(58)**	11	(58)	15	(79)
**Cancer Diagnosis**																										
Breast cancer (n = 35)	13	(38)	13	(38)	9	(27)	2	(6)	16	(47)	18	(51)	15	(43)	10	(29)	13	(37)	15	(43)	10	(29)	17	(49)	21	(60)
Other cancers (n = 36)	11	(32)	6	(18)	9	(27)	1	(3)	17	(50)	20	(56)	16	(44)	11	(31)	12	(33)	13	(36)	16	(44)	20	(56)	20	(56)
**Cancer Stage**																										
Local (Stages I–III) (n = 44)	18	(42)	15	(35)	12	(28)	1	(2)	21	(49)	27	(61)	21	(48)	15	(34)	19	(43)	**23**	**(52)**	17	(39)	26	(59)	**30**	**(68)**
Metastatic (Stage IV) (n = 24)	5	(23)	3	(14)	6	(27)	2	(9)	11	(50)	10	(41)	9	(38)	6	(25)	6	(25)	**4**	**(17)**	8	(33)	10	(42)	**10**	**(42)**
**Cancer Side Effects**																										
≤2 side effects (n = 41)	15	(39)	12	(31)	7	(18)	1	(3)	**15**	**(39)**	21	(51)	14	(34)	11	(27)	15	(37)	18	(44)	15	(37)	24	(59)	20	(49)
>2 side effects (n = 30)	9	(31)	7	(24)	11	(38)	2	(7)	**18**	**(62)**	17	(57)	17	(57)	10	(33)	10	(33)	10	(33)	11	(37)	13	(43)	21	(70)
**Perceived Exercise Benefit: General Health**																										
No/Unsure (n = 30)	**4**	**(14)**	4	(14)	4	(14)	1	(4)	**8**	**(24)**	**5**	**(17)**	**9**	**(30)**	**4**	**(13)**	**5**	**(17)**	**8**	**(27)**	**7**	**(23)**	**8**	**(27)**	**9**	**(30)**
Yes (n = 38)	**20**	**(53)**	15	(40)	14	(37)	2	(5)	**25**	**(66)**	**33**	**(85)**	**22**	**(56)**	**17**	**(43)**	**20**	**(51)**	**20**	**(51)**	**19**	**(49)**	**28**	**(72)**	**32**	**(82)**
**Perceived Exercise Benefit: Cancer-Related**																										
No (n = 19)	**2**	**(12)**	**0**	**(0)**	**0**	**(0)**	0	(0)	**3**	**(18)**	**3**	**(15)**	**5**	**(25)**	**0**	**(0)**	**2**	**(10)**	**2**	**(10)**	**0**	**(0)**	**4**	**(20)**	**3**	**(15)**
Yes (n = 36)	**19**	**(53)**	**16**	**(44)**	**15**	**(42)**	3	(8)	**23**	**(64)**	**29**	**(81)**	**20**	**(56)**	**15**	**(27)**	**19**	**(53)**	**22**	**(61)**	**19**	**(53)**	**26**	**(72)**	**31**	**(86)**
**Current PA Goals**																										
No increase in PA (n = 24)	**3**	**(14)**	3	(14)	3	(14)	1	(5)	**6**	**(27)**	**7**	**(28)**	9	(36)	4	(16)	**3**	**(12)**	**5**	**(20)**	6	(24)	**8**	**(32)**	**8**	**(32)**
Increase in PA (n = 42)	**21**	**(50)**	16	(38)	15	(36)	2	(5)	**27**	**(64)**	**31**	**(74)**	22	(52)	17	(41)	**22**	**(52)**	**23**	**(55)**	20	(48)	**28**	**(67)**	**33**	**(79)**
**Current Exercise Motivators**																										
≤3 motivators (n = 38)	**4**	**(11)**	**3**	**(9)**	**5**	**(14)**	0	(0)	**12**	**(34)**	-	(-)	-	(-)	-	(-)	-	(-)	-	(-)	-	(-)	-	(-)	-	(-)
>3 motivators (n = 33)	**20**	**(61)**	**16**	**(49)**	**13**	**(39)**	3	(9)	**21**	**(64)**	-	(-)	-	(-)	-	(-)	-	(-)	-	(-)	-	(-)	-	(-)	-	(-)
**Exercise Facilitating Factors**																										
≤1 facilitator required (n = 41)	-	(-)	-	(-)	-	(-)	-	(-)	-	(-)	**15**	**(37)**	**12**	**(29)**	**7**	**(17)**	**7**	**(17)**	**8**	**(20)**	**9**	**(22)**	**16**	**(39)**	**14**	**(34)**
>1 facilitator required (n = 30)	-	(-)	-	(-)	-	(-)	-	(-)	-	(-)	**23**	**(77)**	**19**	**(63)**	**14**	**(47)**	**18**	**(60)**	**20**	**(67)**	**17**	**(57)**	**21**	**(70)**	**27**	**(90)**
**Interest in Community Exercise Program**																										
No/Unsure (n = 31)	**5**	**(18)**	5	(18)	4	(14)	1	(4)	**6**	**(21)**	**7**	**(23)**	10	(32)	**4**	**(13)**	**6**	**(19)**	**6**	**(19)**	**5**	**(16)**	**9**	**(29)**	**11**	**(36)**
Yes (n = 38)	**18**	**(47)**	13	(34)	14	(37)	2	(5)	**27**	**(71)**	**31**	**(82)**	21	(55)	**17**	**(45)**	**19**	**(50)**	**21**	**(55)**	**21**	**(55)**	**28**	**(74)**	**29**	**(76)**

^1^ Bolded values represent significant differences between subgroups.

## Data Availability

Data available on request due to restrictions.

## Supplementary Materials

The following are available online at https://www.mdpi.com/article/10.3390/curroncol28040276/s1, Questionnaire S1: The Rural Exercise for Cancer Patients and Survivors (RECaPS) Study.
