# Supplementary material for: Characterizing the Exercise Behaviour, Preferences, Barriers, and Facilitators of Cancer Survivors in a Rural Canadian Community: A Cross-Sectional Survey
Source: Curr Oncol. 2021 Aug 19;28(4):3172–87. doi: 10.3390/curroncol28040276 (PMC8395505; doi:10.3390/curroncol28040276)
Supplement: Supplementary file 1 [file curroncol-28-00276-s001.zip › curroncol-1342244-supplementary.pdf]

LaurentianUniversity
UniversitéLaurentienne

MUSKOKA ALGONQUIN HEALTHCARE

The Rural Exercise for Cancer Patients and Survivors (RECaPS) Study

Investigators:

Scott C. Adams, PhD, CSEP-CEP[1,2]; Jenna Smith-Turchyn, PhD, PT[1]; Helen Dempster, MD, CCFP, MSc, BScEng[3,4]; Rich Trenholm, MD, CCFP, CFPC, MSc[3,4]; Deanna Lavigne, RKin, BScKin; Lisa Allen, PhD[3,4]; Krista Hailstone, B.Comm.[3,4]; Catherine Sabiston, PhD[1]; Lindsay MacMillan, MD, CCFP[3,4]

[1] – University of Toronto; [2] – Princess Margaret Cancer Centre; [3] – Northern Ontario School of Medicine; [4] – Muskoka Algonquin Health Care

Instructions:

Thank you for agreeing to participate in this study. In this questionnaire, we are going to ask you a series of questions about yourself. This part of the questionnaire collects important information regarding the characteristics and needs of people diagnosed with cancer and living in the greater Huntsville area.

Many of the questions ask you about your current health and health history, and some may be viewed as personal. It is important to answer as many of these questions as possible; however, you may leave questions blank if you are uncomfortable answering them.

Please Note: All responses are completely confidential and will never be used in any way that could link them to you. By returning this questionnaire, you are providing consent for the study Investigators to use your data anonymously.

If you have any questions about completing the questionnaire, please contact our Exercise Lead (Deanna Lavigne) or our Research Coordinator (Lisa Allen) at 705-789-2311 x 2712 or therecapsstudy@gmail.com.

DEMOGRAPHICS

1. Age: ______

2. Location of Residence (in relation to downtown Huntsville):

downtown	_____	10-minute drive	_____	30-minute drive	_____
5-minute drive	_____	20-minute drive	_____	40+ minute drive	_____

3. Current Marital Status:

Never Married	_____	Married	_____	Common Law	_____
Separated	_____	Widowed	_____	Divorced	_____

4. Education (Please check highest level attained):

Some High School	_____	Completed High School	_____	Some University/College	_____
Completed Univ/Coll	_____	Some Graduate School	_____	Completed Grad School	_____

5. Annual Family Income:

< $20,000	_____	$20-39,999	_____	$40-59,999	_____
$60-79,999	_____	$80-99,999	_____	> $100,000	_____

6. Current Employment Status:

Disability	_____	Retired	_____	Part Time	_____
Full Time	_____	Sick Leave	_____		

7. What is your primary ethnic origin or race (please circle)?

White Black Hispanic Asian Aboriginal Other ______________________________

8. Which of the following best describes your current smoking status?

Never Smoked _____ Ex-Smoker _____ Current Smoker _____

9. Has a doctor or nurse ever told you that you had any of the following conditions? (check all that apply):

High blood pressure	____	No	____	Yes	High cholesterol	____	No	____	Yes
Heart attack	____	No	____	Yes	Stroke	____	No	____	Yes
Emphysema	____	No	____	Yes	Chronic bronchitis	____	No	____	Yes
Diabetes	____	No	____	Yes	Other cancer	____	No	____	Yes
Angina (chest pains)	____	No	____	Yes	Arthritis	____	No	____	Yes

Any other long-term health condition? ____

10. In the past month, was your ability to exercise limited by a health condition, injury, or disability?

1	2	3	4	5
Not at all	A little	Somewhat	Quite a lot	Completely

11. Are you currently taking any medications for health problems? (e.g., for anxiety, depression, blood pressure, constipation, pain, to help with sleep, etc.)

What is the medication?	What is it for? (e.g., blood pressure, anxiety)
1. ______________________	______________________
2. ______________________	______________________
3. ______________________	______________________

Others? ____

12. Have you experienced any recent weight loss or gain? (please check)

12a. Weight loss? _____ Yes _____ No **12b. Weight gain?** _____ Yes _____ No

If **Yes**, how much? ______________________ If **Yes**, how much? ______________________

13. Please rate the average level of fatigue you currently experience within a typical day.

0	1	2	3	4	5	6	7	8	9	10
None		Mild			Moderate			Severe		Extreme

Cancer History

1. What type of cancer were you diagnosed with (most recent)?

____

2. When were you diagnosed with this type of cancer (month/year)?

____

3. Did your cancer involve the lymph nodes? (please check)

_____ Yes _____ No _____ Unsure

4. Was your cancer described as "localized" (confined to one area of the body) or "metastasized" (spread to other parts of the body)?

_____ Localized _____ Metastasized _____ Unsure

5. If your cancer was described as metastasized, where else in your body was it? (check all that apply)

_____ Lung _____ Lymph nodes _____ Brain _____ Liver

_____ Bone _____ Other (Please specify: ______________________) _____ Unsure

6. Did your treatment involve surgery? (please check)

_____ Yes _____ No

If **Yes**, please provide the following details of your surgery or surgeries:

6a. How many surgeries: ______________________

6b. Area(s) of your body: ______________________

6c. Surgery dates [month(s)/year(s)]: ______________________

7. Did your treatment involve radiation therapy? (please check)

_____ Yes _____ No

If **Yes**, please provide the following details of your radiation therapy:

7a. How many treatments: ______________________

7b. Area(s) of your body treated: ______________________

7c. What was the date of your last treatment (month/year): ______________________

8. Did your treatment include chemotherapy? (please check)

_____ Yes _____ No

If **Yes**, please provide the following details of your chemotherapy treatment:

8a. What type(s) of chemotherapy did you receive? ______________________

8b. How many treatments (of each type) did you receive? ______________________

8c. What was the date of your last treatment (month/year)? ______________________

9. Did you receive any other type of treatment? (please check)

_____ Yes _____ No

If **Yes**, please provide the following details of your other treatments:

Additional Treatment #1

9a. What type of treatment? ______________________

9b. How many treatments did you receive? ______________________

9c. What was the date of your last treatment? ______________________

Additional Treatment #2

9d. What type of treatment? ______________________

9e. How many treatments did you receive? ______________________

9f. What was the date of your last treatment? ______________________

10. What is the current status of your cancer treatments? (please check)

_____ I am not currently receiving any cancer treatments.

_____ I am **currently receiving cancer treatment**.

If **currently receiving cancer treatment**, what treatment(s)? ______________________

11. Have you ever had a recurrence of your cancer? (please check)

_____ Yes _____ No _____ Unsure

12. Is this the only type of cancer you have been diagnosed with? (please check)

_____ Yes   _____ No

13. If No, please list the other types of cancer, dates of diagnosis (month/year), and types/details of the treatments received in the space below:

____
____
____
____
____
____

14. Do you have any complications due to treatment? (please check)

_____ Yes   _____ No

If **Yes**, please list (e.g., infections, low blood counts, muscle/bone/joint pain):

____
____

RECaPS Needs Assessment

PART 1: Please answer the following questions:

1. **During a typical 7-day period in the last month, how often do you take part in the following kinds of exercise during your free time and for how long?**

   STRENUOUS EXERCISE - Heart beats rapidly
   (e.g. running, jogging, hockey, football, soccer, squash, basketball, vigorous swimming or bicycling)

   Number of times per week ______________________

   Average duration of each session (minutes) ______________________

   MODERATE EXERCISE - Not exhausting
   (e.g. fast walking, easy bicycling, easy swimming, downhill skiing)

   Number of times per week ______________________

   Average duration of each session (minutes) ______________________

MILD EXERCISE - Minimal effort
(e.g. yoga, taking the stairs, bowling, easy walking)

Number of times per week ____________________

Average duration of each session (minutes) ____________________

RESISTANCE / STRENGTH EXERCISE
(e.g., lifting weights, push ups, sit ups, resistance bands)

Number of times per week ____________________

Average duration of each session (minutes) ____________________

2. In general, I engage in exercise (check any that apply):

- ☐ Alone
- ☐ With an organized group
- ☐ At a gym or community centre
- ☐ At a medical facility
- ☐ With family or friends
- ☐ At home
- ☐ Outside

3. Describe your physical activity level during the following times:

Prior to your cancer diagnosis

☐ Not active at all ☐ A little active ☐ Somewhat active ☐ Very active

During your cancer treatment

☐ Not active at all ☐ A little active ☐ Somewhat active ☐ Very active ☐ Not applicable

After your cancer treatment

☐ Not active at all ☐ A little active ☐ Somewhat active ☐ Very active ☐ Not applicable

4. What were the barriers to exercise participation *during* your cancer treatment? (check all that apply)

- ☐ Cost
- ☐ Time
- ☐ Child care
- ☐ Unaware of any available exercise program
- ☐ Distance to exercise program
- ☐ Transportation
- ☐ Physical side effects (if checked, please list): ____________________

____

- ☐ Other (please describe): ____________________

____

5. What were the barriers to exercise participation *after* your cancer treatment? (please check all that apply)

- ☐ Cost
- ☐ Time
- ☐ Child care
- ☐ Physical side effects (if checked, please list): ____________________
- ☐ Unaware of any available exercise program
- ☐ Distance to exercise program
- ☐ Transportation
- ☐ Other (please describe): ____________________

6. Were you informed about the benefits of exercise by your health care team?

☐ Yes ☐ No

7. If yes, which member of your team discussed exercise with you?

- ☐ Surgeon
- ☐ Radiation Oncologist
- ☐ Physiotherapist
- ☐ Family Doctor
- ☐ Other (please describe): ____________________
- ☐ Medical Oncologist
- ☐ Nurse
- ☐ Kinesiologist

8. Do you feel participation in an exercise program would be beneficial for you at this time?

☐ Yes ☐ No ☐ Unsure

9. Please explain your response to question #8 (why do you feel this way?).

____

____

____

10. How confident are you that you could exercise three to five times per week for 30-60 minutes during each session? (please circle)

Not confident ... Extremely confident

1 2 3 4 5 6 7 8 9 10

11. Do you currently have any side effects of treatment?

- ☐ Yes
- ☐ No

12. If you answered yes to question 11, please check all of the side effects that apply:

- ☐ Fatigue
- ☐ Lymphedema
- ☐ Depression
- ☐ Anxiety
- ☐ Nausea / Vomiting
- ☐ Loss of Appetite
- ☐ Pain (if checked, please list location(s)): ______________________
- ☐ Decreased strength (if checked, please list location(s)): ______________________
- ☐ Decreased range of motion (if checked, please list location(s)): ______________________
- ☐ Other (if checked, please list): ______________________

13. If you answered yes to question 11, please describe how these side effects have affected your function:

- ☐ Unable to return to work (if checked, list occupation): ______________________
- ☐ Unable to return to important activities at home
- ☐ Unable to return to important activities in the community
- ☐ No change in function
- ☐ Other (please describe): ______________________

14. What are your cancer-related concerns at this time?

____

____

____

15. Do you feel involvement in an exercise program could help these concerns?

- ☐ Yes
- ☐ No

16. Which of the following best describes you right now? I want to…

- ☐ Increase my current level of physical activity

- ☐ Maintain my current level of physical activity
- ☐ Decrease my current level of physical activity
- ☐ I do not currently have goals related to my physical activity

17. What would motivate you to participate in an exercise program at this time? (check all that apply)

- ☐ Increase fitness level
- ☐ Increase social interaction
- ☐ Lose weight
- ☐ Prevent cancer recurrence
- ☐ Reduce stress
- ☐ Prevent disease
- ☐ Improve ability to do things that matter to me (e.g., social activities with family and friends)
- ☐ Increase strength
- ☐ Other (please describe): ______________________

18. What would help you to successfully participate in exercise at this time? (check all that apply)

- ☐ Access to a gym
- ☐ Social support
- ☐ Access to exercise equipment
- ☐ Transportation assistance
- ☐ Information from a qualified exercise professional
- ☐ Other (please list): ______________________

19. Do you currently have access to a gym at your home?

☐ Yes ☐ No

20. Are you currently a member of a gym or fitness centre?

☐ Yes ☐ No

21. Would you like to participate in the RECaPS exercise program at this time or within the next two years?

☐ Yes ☐ No ☐ Unsure

PART 2: If you <u>declined to participate</u> in the RECaPS Exercise Program, could you please answer the following questions:

1. **Why did you decline to participate in the RECaPS exercise program?**

____
____
____
____
____
____
____
____
____
____

2. **What are your current barriers to participating in this study?**

- ☐ Cost
- ☐ Time
- ☐ Child care
- ☐ Unaware of any available exercise program
- ☐ Distance to exercise program
- ☐ Transportation
- ☐ Physical side effects (if checked, please list): ______________________
____
- ☐ Other (please describe): ______________________
____

3. **How could the RECaPS exercise program be modified to help you overcome the barriers you listed above?**

____
____
____
____
____
____
____
